# Early wound healing of laser in situ keratomileusis–like flaps after treatment with human corneal stromal stem cells

**DOI:** 10.1016/j.jcrs.2015.09.023

**Published:** 2016-02

**Authors:** Siân R. Morgan, Erin P. Dooley, Christina Kamma-Lorger, James L. Funderburgh, Martha L. Funderburgh, Keith M. Meek

**Affiliations:** From the Structural Biophysics Research Group and Cardiff Institute of Tissue Engineering and Repair (Morgan, Dooley, Kamma-Lorger, Meek), School of Optometry and Vision Sciences, Cardiff University, Cardiff, United Kingdom; the Department of Ophthalmology (J.L. Funderburgh, M.L. Funderburgh), University of Pittsburgh, Pittsburgh, Pennsylvania, USA

## Abstract

**Purpose:**

To use a well-established organ culture model to investigate the effects of corneal stromal stem cells on the optical and biomechanical properties of corneal wounds after laser in situ keratomileusis (LASIK)–like flap creation.

**Setting:**

School of Optometry and Vision Sciences, Cardiff University, Cardiff, Wales, United Kingdom.

**Design:**

Experimental study.

**Methods:**

The LASIK-like flaps were produced in sheep corneas. The flap beds were treated with corneal stromal stem cells and were then replaced and allowed to heal for different periods of up to 3 weeks in organ culture. The optical transmission of the cornea, the force required to detach the flap, and the presence of myofibroblasts near the flap bed were measured.

**Results:**

Corneal stromal stem cell–treated flap beds were statistically significantly more transparent after 3 weeks in culture than the untreated controls. At 3 weeks, the mean force necessary to detach the flap was more than twice the force required for the respective control samples. Concurrently, there were 44% activated cells immediately below the flap margin of the controls compared with 29% in the same region of the corneal stromal stem cell–treated flaps.

**Conclusions:**

In this system, the presence of corneal stromal stem cells at the wound margin significantly increased the adherence of LASIK-like flaps while maintaining corneal transparency. It is postulated that this is achieved by the deposition of extracellular connective tissue similar to that found in the normal cornea and by the paucity of activated keratocytes (myofibroblasts), which are known to scatter a significant amount of the incident light.

**Financial Disclosure:**

No author has a financial or proprietary interest in any material or method mentioned.

Clinical procedures that improve the refractive state of the eye are increasingly used to improve vision. Laser in situ keratomileusis (LASIK) is the most common elective operation, with more than 35 million procedures performed worldwide by 2010.^1^ In addition, there are corneal injuries that account for a small but significant fraction of ocular trauma and that require immediate assistance; these include burns (chemical and thermal) and subepithelial abrasions.^2^, ^3^ In general, the outcome of surgical or accidental corneal injury is a reduction in biomechanical strength and transparency, with incomplete adherence of the affected tissue after extracellular matrix (ECM) remodeling. These changes depend on the type of injury, and hence on the type of connective tissue, and other materials deposited as well as on changes to the refractive index of corneal cells after activation with inflammatory growth factors and cytokines.^4^, ^5^

The specialized order of the cornea's stromal collagen governs the biomechanical and transparent properties of the tissue.^6^ When an injury is incurred, this precise collagenous arrangement is altered. The initial objective of the wound-healing cascade is to rapidly barricade the wound and prevent the invasion of foreign bodies or infections.^7^ The impaired epithelium releases cytokines, causing swelling and inflammation that can lead to changes in corneal hydration. Activated corneal keratocytes heal the wound by producing collagen and other ECM materials; however, this can lead to a reduction in transparency. Furthermore, fibroblasts (activated keratocytes) and myofibroblasts (more highly activated fibroblasts that are capable of contraction) show significantly reduced levels of crystallin proteins,^4^, ^8^ and this has been found to correlate with a marked increase in light scattering or “corneal haze.”^9^ In rabbits, it takes at least 6 months for the remodeling to stop and the cells to undergo apoptosis or return to homeostasis.^10^

In procedures such as LASIK in which a hinged flap is created in the stromal bed,^11^ limited wound healing occurs by means of an epithelial plug around the cut margins and the deposition of minimal fibrotic material along the bed of the wound.^12^ Laser in situ keratomileusis flaps are known to be inadequate healers, retaining only 2% to 28% of their tensile strength for more than a decade after surgery.^13^ Therefore, ectasia as well as partial and complete flap detachments are common long-term postoperative challenges.^14^, ^15^, ^16^, ^17^ The fragility of the flap margin also means that the interface is prone to opening, and this introduces the risk for exposing the patient to infection from opportunistic organisms such as viruses and bacteria. For these reasons, increasing flap adhesive strength while maintaining transparency is a clinical goal. In previous studies, corneal fibroblasts, crosslinking, and fibrinogen-based glues have been used in an attempt to increase flap strength in in vitro models.^18^, ^19^

New work has involved a range of topical, engineered, and cellular treatments to increase wound healing and decrease scar formation. Recently, stem cells isolated from adult human corneal stroma were earmarked as having the potential as a stem cell–based treatment for corneal opacity.^20^ These cells, referred to as corneal stromal stem cells, have been shown to have the ability to remodel stromal ECM into a tissue essentially indistinguishable from that of wild-type matrix, and they maintain the ability to produce this ECM even after extensive expansion in vitro.^20^, ^21^ In the current study, we examined the ability of these cells to improve corneal wound healing and hence increase the adherence strength of LASIK-like flaps while maintaining clarity in an ovine corneal model.

## Materials and methods

### Organ Culture and Cell Treatment

Ninety whole ovine corneas were obtained from a local abattoir and surgically wounded by introducing a corneal flap of midstromal depth using a microkeratome (Hansatome, Bausch & Lomb). Only healthy eyes with clear/transparent corneas were selected for wounding. The stromal bed beneath the flap was not ablated as performed in traditional LASIK procedures; however, the wounds are referred to as LASIK-like. The 90 wounded eyes were divided into 2 groups; that is, wounded controls and those to be treated with human corneal stromal stem cells.

Human corneal stromal stem cells were provided by the University of Pittsburgh, School of Medicine, Pittsburgh, Pennsylvania, USA. They were shipped on ice, and on arrival they were incubated overnight at 37°C in a 5% carbon dioxide (CO_2_) incubator to ensure cell attachment. They were then washed with sterile phosphate-buffered saline (PBS) before being treated with trypsin (TrypLE, Life Technologies) for 10 minutes at 37°C. Last, the cells were gently centrifuged at 4°C and resuspended in medium (Medium 199, Life Technologies Corp.) at 6.5 cells/mL × 10^4^ cells/mL.

Forty-five corneas were treated with human corneal stromal stem cells. The LASIK-like flaps were raised using a sterile pipette tip, and 10 μL of the 6.5 cells/mL × 10^4^ cells/mL suspension was carefully applied beneath each flap. The flap was then repositioned using a pipette tip. The corneas were dissected out, leaving a scleral rim of approximately 2.0 mm, and cultured as previously described^18^, ^22^, ^23^ for up to 3 weeks. A preparation of agar–gelatin support gel was introduced into the posterior endothelial cavity of each cornea to the level of the limbal ring, and each cornea was inverted into a sterile petri dish. Gibco M199 medium (Invitrogen Corp.) containing amphotericin B (Fungizone) and antibiotics was added to each dish up to the limbal area to preserve the corneas during the culture period. The dishes were then transferred to a sterile CO_2_ incubator to incubate for the required culture time period. Two hundred microliters of medium were applied to each cornea twice every 24 hours throughout the duration of the experiment to keep the corneal surface moist and to prevent bacterial or fungal infection. The culture medium in each dish was replaced with fresh medium every 4 days during the culture period. Forty-five corneas acted as wounded controls without the application of cells and were processed for culture as above. The corneas were removed from culture at 1-week, 2-week, and 3-week timepoints (14 treated and 14 control corneas at 1 and 2 weeks and 17 treated and 17 control corneas at 3 weeks). Because of the nature of the organ culture process, not all corneas introduced into culture survived for experimentation. After the culture period, the corneas were immersed in dextran 8% solution (in medium) at 37°C overnight to restore homeostasis before assessment of transparency, flap adherence, and mechanical strength.

### Evaluation of Corneal Transparency

Transparency was evaluated by taking spectrophotometric measurements across the visible spectrum. A spectrophotometer (SP8-100 UV/VIS, Pye Unicam Ltd.) was used to measure the transparency of the central flap region. To minimize possible effects in light transmission caused by differences in stromal hydration and thickness, central corneal pachymetry (Pachmate DGH55, DGH Technology, Inc.) was performed on all samples. Only corneas with a thickness of less than 850 μm were included. The corneas were rinsed in PBS to remove the dextran and introduced to a purpose-built chamber with 2 flat machine-polished glass windows. The cornea's natural curvature was maintained by clamping the scleral rim in the sample holder and injecting silicone oil (200/5cS, Dow Corning) behind it. Corneas that were too small to allow adequate clamping were excluded and retained for mechanical strength or immunohistochemical assessment. To maintain a uniform refractive index and limit light scatter, silicone oil was also injected into the front chamber of the holder. The sample holder was then positioned into the spectrophotometer in such a way that light passed through the center of the flap in the anterior–posterior direction. The 1.0 mm beam of white light was passed through a series of filters to produce monochromatic light. A transmission spectrum was then measured first for the chamber filled with silicone oil to act as the blank and then for each cornea at 10 nm intervals in the range of 400 to 700 nm. The measurements were repeated 3 times for each sample. Further readings were taken toward the periphery of the tissue to ensure the reliability of the transparency readings, and the transparency decreased with the greater thickness of the peripheral tissue. The values were normalized by dividing the blank values into the respective sample values and multiplying by 100 to obtain the percentage transmittance of light across the wavelengths.

### Assessment of Flap Mechanical Adherence

The extent of flap adhesion in both corneal stromal stem cell–treated and untreated samples was measured using a vertical extensometer set to perform a “pull to break” test (Lloyd Instruments Ltd.). Cardboard strips of dimensions 0.25 cm × 2.5 cm were cut and adhered using cyanoacrylate adhesive to the middle of the anterior side and posterior side of each cornea. The strips were clamped opposite each other so that the force would pull along the *y*-axis. The force that was required to separate the flap from the underlying stromal bed (referred to as the first breakpoint) was recorded using the Nexygen 4.1 software package (Lloyd Instruments Ltd.).

### Immunohistochemistry

Cell phenotype of activated fibroblasts during the healing process was examined by anti-α-smooth muscle actin (α-sma) immunostaining at each of the 3 culture timepoints. The corneal samples were stored in paraformaldehyde 4% (at 4°C) for 24 hours and then wax embedded. Wax sections (10 μm thick) cut with a microtome (Leica Microsystems GmbH) were mounted on Histabond adhesive slides (Utech Products, Inc.) and left overnight to adhere. As a consequence, tissue sections were dewaxed using xylene and rehydrated in decreasing concentrations of ethanol. The sections were then treated with goat serum (Sigma-Aldrich Co.) in PBS (1:4 vol/vol) for 30 minutes at room temperature. The primary antibody, anti-α-sma mouse monoclonal antibody (Sigma-Aldrich Co.), in PBS–0.2% bovine serum albumin (1/200) was applied to the sections and left to incubate overnight at 4°C. The following day, the sections were incubated with the secondary antibody Alexa Fluor 488 goat anti-mouse immunoglobulin G (Invitrogen Corp.) (1/1000) in PBS along with 3 μL of Hoescht 33342 (Invitrogen Corp.) for 2 hours in the dark at room temperature and mounted with Hydromount (National Diagnostics). Finally, all slides were imaged using a Leica 6000 fluorescent microscope with 4′,6-diamidino-2-phenylindole dihydrochloride (blue) and fluorescein isothiocyanate (green) filter sets for detection of Hoescht 33342-bound and Alexa Fluor 488-bound α-sma, respectively, at ×20 magnification. Average total cell counts were obtained by measuring the total number of cells in a set field of view and were averaged over 6 fields of view per section (6 tissue sections per sample). The field of view was a region of the image taken at ×20 magnification of a 10 μm thick section (n = 3 treated and n = 3 control corneas at each timepoint).

### Statistical Analysis

For direct comparisons within and between timepoints, the normalized transmitted intensity measurements in the middle of the visible spectrum (550 nm) and the breakpoint data were examined statistically by *t* tests or analysis of variance (ANOVA) and post hoc least-significant-difference testing using Statistica software (version 7.1, Statsoft, Inc.).

## Results

### Transparency

**Figure 1:**
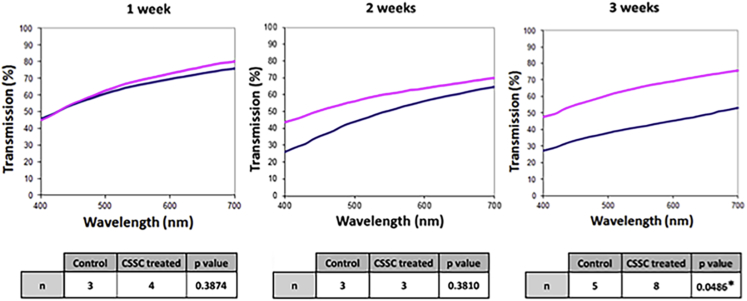
Transparency results at 1, 2, and 3 weeks for corneal stromal stem cell–treated samples (*pink*) and wounded control samples (*blue*) show that the transparency of the corneal stromal stem cell–treated corneas at 550 nm was significantly higher than that of the control corneas by 3 weeks (* = statistically significant, unpaired *t* tests [at 550 nm]; CSSC = corneal stromal stem cell).

The transparency of the samples could be measured at all organ culture time periods, verifying the integrity of the culture technique and its capacity to limit corneal swelling (Figure 1). At 0 weeks, all corneas were visually clear; however some, in particular in the control group, became cloudier with incubation time. Spectrophotometry showed that the difference between the control group and the treated group became statistically significant only after 3 weeks. The thickness of the control samples at 2 and 3 weeks was on average greater than that of the treated samples at these timepoints and the 1-week control samples; however, this increase in thickness was not significant (Table 1). Therefore, it can be said that the differences in transparency observed between control corneas and treated corneas were a direct result of the corneal stromal stem cell application as opposed to being attributed to the culture model.

### Mechanical Adhesion of the Flap

**Figure 2:**
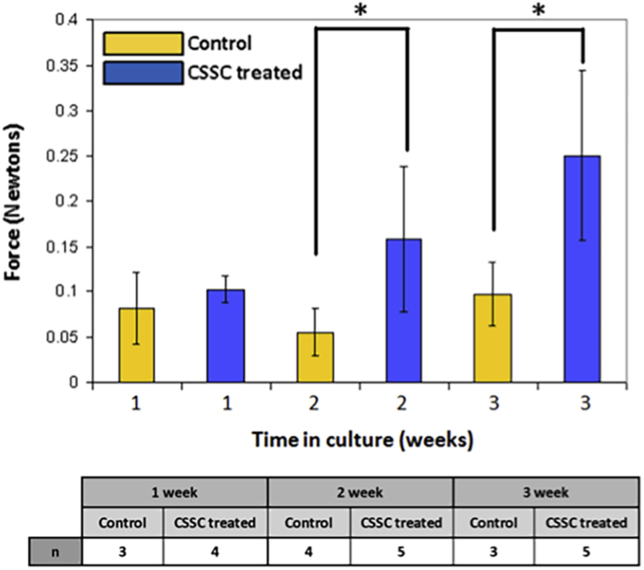
Mechanical assessment of control and corneal stromal stem cell–treated flap adherence. The mean flap detachment force was significantly greater in the corneal stromal stem cell–treated corneas than in the controls at week 2 (*P* = .044). By the third week culture point, the mean force was more than twice the level of the control corneas (*P* = .038) (* = statistically significant, unpaired *t* tests; CSSC = corneal stromal stem cell).

The mean force required to detach the flap at the 1-week timepoint appeared to be elevated in response to cell treatment compared with controls; however, this was not significant with the sample numbers used (*P* > .05, ANOVA with Tukey post hoc least-significant-difference testing) (Figure 2). The mean flap detachment force decreased in control samples at 2 weeks compared with 1 week and then increased again after 3 weeks; however, statistical analysis showed no significant differences in mean force values between the 3 timepoints (*P* > .05, 1-way ANOVA with Tukey post hoc tests). The flap strength of the corneal stromal stem cell–treated samples gradually increased as the duration of the culture increased; however, the difference between control samples and treated samples became significant only after 3 weeks in culture. At the 3-week timepoint, the mean force necessary to detach the flap was more than twice the force required for the respective control samples.

### Myofibroblast Expression

Immunohistochemistry indicated a marked increase in the total number of cells in the control corneas and cell-treated corneas between 1 week and 2 weeks, with treated corneas having larger cell counts than control corneas at both timepoints. After 3 weeks in culture, there was a detectable decrease in the stromal cell number in the keratectomy wound region in both control corneas and treated corneas. Using the cell count at 1 week as a baseline, treated tissue cell numbers diminished to below this value, whereas control tissue cell numbers, despite a pronounced decrease after 3 weeks, remained elevated.

**Figure 3:**
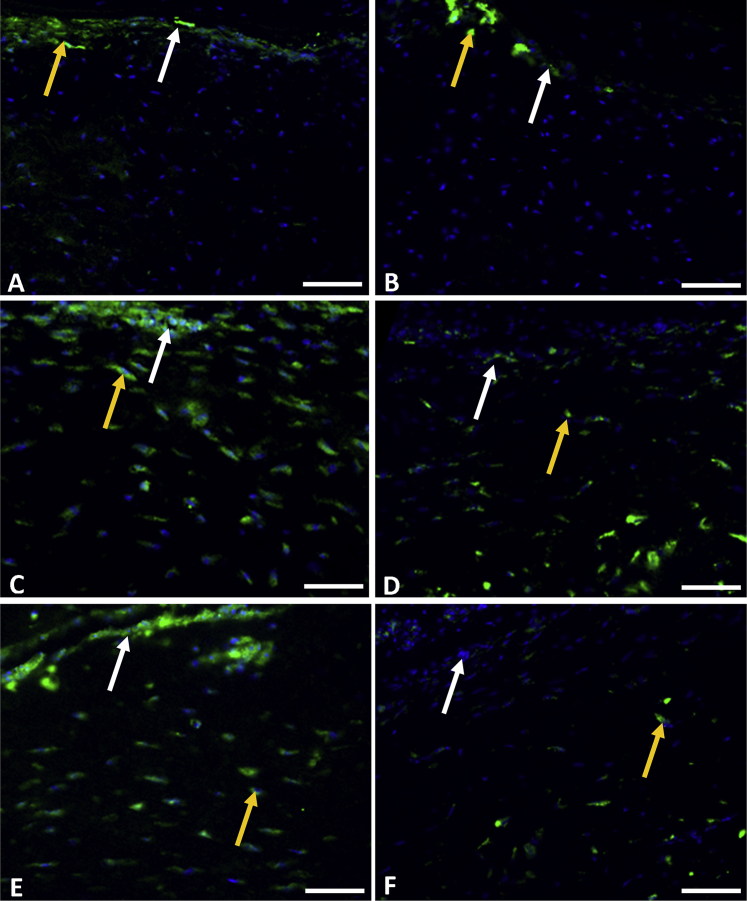
Immunohistochemistry results for α-sma expression in LASIK-like flap beds of control corneas (*left*) and corneal stromal stem cell–treated corneas (*right*) after 1 week (*A* and *B*), 2 weeks (*C* and *D*), and 3 weeks (*E* and *F*) in culture. Treated corneas showed fewer α-sma positive cells than control corneas at all 3 timepoints (*yellow arrows*). (green = α-sma; blue = Hoechst cell nuclei). The *white arrows* indicate the flap bed (original magnification ×20; calibration bars = 50 μm).

Figure 3 shows examples of α-sma immunolabeling detected in control samples and treated samples at each culture timepoint. The extent of α-sma staining in control tissue and corneal stromal stem cell–treated tissue was comparable and was largely restricted to the stromal flap bed. After 2 weeks and 3 weeks in culture, the control samples had an increase in α-sma staining along the flap bed and deeper into the tissue below the flap bed (Table 2). In the corresponding regions of corneal stromal stem cell–treated tissue, the computed α-sma activation at these timepoints was lower. These α-sma–expressing cells were predominately located deeper in the tissue below the flap bed (Figure 3, *D* and *F*). The mean percentage activation was consistently lower in treated samples than in control samples, without substantial increases, and this correlated with the invariable transparency observed through the culture periods in these corneas.

## Discussion

In this study, the organ culture model as outlined by Mi et al.,^18^ Foreman et al.,^22^ and Kamma-Lorger et al.^23^ was used to assess LASIK-like wound healing after the application of stem cells from the human corneal stroma. This organ culture method is favored over simple cell culture for observing wound healing because of its capacity to imitate corneal structure, cellular interactions, and wound healing with accuracy.^24^

The increase in flap strength measured for corneal stromal stem cell–treated corneas can be attributed to the demonstrated potential for corneal stromal stem cells to produce and organize abundant connective tissue including collagens type I, V, and VI and keratan sulfate proteoglycans that are located only in the cornea.^25^, ^26^, ^27^ When corneal stromal stem cells are introduced into corneas in vivo, they also adopt a keratocyte phenotype and secrete human stromal matrix components, replacing disorganized or scar tissue with organized stromal tissue indistinguishable from that of native cornea.^20^, ^21^ Based on these previous studies, we speculate that in our current experiments, remodeling of the connective tissue on either side of the keratectomy wound by corneal stromal stem cells was in progress, leading to the increased wound strength at 3 weeks and eventually full healing of the flap to the rest of the cornea. Larger in vitro and in vivo studies performed for extended periods will be required to confirm this hypothesis. Tests at the edge and central regions of the flap will also be necessary.

By 3 weeks, the transparency of corneal stromal stem cell–treated corneas was significantly higher than that of untreated control corneas. At this timepoint, there was no significant difference in corneal thickness between control samples and the treated samples; however, at this time, the corneal stromal stem cells begin to produce abundant corneal ECM.^27^ Tissue secreted by corneal stromal stem cells is distinct from that produced in similar conditions by corneal fibroblasts,^28^ cells that typically populate corneal wounds. Fibroblasts are less abundant than myofibroblasts in the wound; however, both secrete matrix components associated with opaque scar tissue.^29^, ^30^ The reduction in myofibroblasts observed in the corneal stromal stem cell–treated corneas argues that, in addition to secreting native corneal matrix, corneal stromal stem cells suppress the number of cells in the region that produce scar tissue.

Human corneal stromal stem cells are located subadjacent to the basement membrane, near the limbal epithelial stem cells.^31^ Corneal stromal stem cells might represent the mesenchymal “niche cells” in this region that help maintain the stem/progenitor character of the epithelial stem cells in vivo.^21^, ^32^ Although data have not demonstrated an active role of these cells in stromal homeostasis, results in previous studies^20^, ^21^ suggest that the default lineage of corneal stromal stem cells is the keratocyte and that introducing them into the stroma initiates this transformation. In damaged stroma, however, these studies showed that corneal stromal stem cells do not simply replace missing ECM but appear to initiate regeneration of stromal tissue by the cells of the host cornea. The ability to induce tissue regeneration is now recognized for mesenchymal stem cells acting on a wide variety of tissues in addition to cornea.^33^, ^34^, ^35^, ^36^, ^37^, ^38^ This process is clearly distinct from wound healing and although it exhibits aspects of embryonic development, it does not exactly recapitulate that process.^39^

The regenerative potential of stem cells might be tied to their immune-modulatory function. Such cells secrete several factors that suppress activation of neutrophils, T-cells, and B-cells but also mediate the phenotype of tissue-resident dendritic cells and macrophages from proinflammatory to regenerative phenotypes.^40^ Exploration of these mechanisms is a matter of active current research; however, definitive molecular mechanisms linking tissue regeneration and immune modulation are speculative at this point.

Understanding the regenerative potential and immunosuppression afforded by corneal stromal stem cells will strengthen the results in this study and support the idea that the application of human corneal stromal stem cells presents a promising approach for improving the clarity of the cornea during wound healing and also for increasing the adherence strength of LASIK corneal flaps. A recent study^36^ showed that such a treatment might even be performed using autologous corneal stromal stem cells. Such an approach would appear to provide a new biological therapy with a high degree of safety.

**What Was Known**
• Laser in situ keratomileusis flaps never heal completely. Only an epithelial plug around the cut edge and what has been termed primitive fibrotic tissue at the flap bed occur.
• Previous attempts to produce better flap adherence resulted in loss of corneal transparency. Thus far, no treatment has been shown to preserve transparency while increasing flap adherence.

**What This Paper Adds**
• Corneal stromal stem cells applied at the LASIK flap margin have the potential to increase flap adherence while retaining corneal transparency.

## Figures and Tables

**Table 1:** Mean corneal thickness measurements for control and treated samples at each culture timepoint.

Time	Mean Corneal Thickness (μm) ± SD	
Control	CSSC Treated
1 week	718 ± 10.3	737 ± 87.5
2 weeks	728 ± 35.1	705 ± 54.6
3 weeks	761 ± 71.1	714 ± 63.4

CSSC = corneal stromal stem cells

There were no significant differences in thickness between groups or across timepoints (all *P* < .05, unpaired *t* tests)

**Table 2:** Mean total cell count and the percentage of activated cells in control and corneal stromal stem cell–treated tissue.

Time/Sample Group	Mean Total Cells (n) ± SD	Mean % Activation
1 week		
Control	176 ± 17.6	24
CSSC treated	204 ± 21.3	19
2 weeks		
Control	223 ± 18.1	48
CSSC treated	227 ± 17.1	30
3 weeks		
Control	191 ± 14.8	44
CSSC treated	186 ± 12.9	29

CSSC = corneal stromal stem cells
